# Supplementary material for: Spin-polarized self-trapped excitons in low-dimensional cesium copper halide
Source: Nat Commun. 2025 Aug 6;16:7264. doi: 10.1038/s41467-025-62704-y (PMC12328795; doi:10.1038/s41467-025-62704-y)
Supplement: Supplementary file 1 — Supplementary Information [file 41467_2025_62704_MOESM1_ESM.pdf]

Supplementary Information

Spin-Polarized Self-Trapped Excitons in Low-Dimensional Cesium Copper Halides

Ruiqin Huang[1], Longbo Yang[2], Feng Yang[1], Yuttapoom Puttisong[3], Qingsong Hu[4], Guixian Li[1], Jingnan Hu[1], Zhaobo Hu[5], Liang Li[1], Jiang Tang[2], Weimin Chen[3],Yibo Han[1*], Jiajun Luo[2*], Feng Gao[3*]

[1]*Wuhan National High Magnetic Field Center and School of Physics, Huazhong University of Science and Technology, Wuhan, 430074, China.*

[2]*Wuhan National Laboratory for Optoelectronics (WNLO) and School of Optical and Electronic Information, Huazhong University of Science and Technology, Wuhan, 430074, China.*

[3]*Department of Physics, Chemistry and Biology (IFM), Linköping University, Linköping, Sweden.*

[4]*Hubei Key Laboratory of Low Dimensional Optoelectronic Materials and Devices, Hubei University of Arts and Science, Xiangyang, 441053, China.*

[5]*School of Chemistry and Chemical Engineering, Jiangxi Provincial Key Laboratory of Functional Crystalline Materials Chemistry, Jiangxi University of Science and Technology, Ganzhou 341000, China*

*E-mail: ybhan@hust.edu.cn (Y. Han), luojiajun@hust.edu.cn (J. Luo), feng.gao@liu.se (F. Gao).

Contents:

Fig. S1: Crystal structure of $Cs_3Cu_2I_5$ upon optical excitation.
Fig. S2: The elemental mapping and EDS spectrum of the sample of $Cs_3Cu_2I_5$.
Fig. S3: Powder XRD patterns of the $Cs_3Cu_2I_5$.
Fig. S4: The crystal structure of $Cs_3Cu_2I_5$.
Fig. S5: PL, PLE and absorption spectra of $Cs_3Cu_2I_5$.
Fig. S6: The excitation wavelength and power dependent PL spectra.
Fig. S7: Band structure of $Cs_3Cu_2I_5$ from first principles calculations.
Fig. S8: The PLQY of $Cs_3Cu_2I_5$.
Fig. S9: *T*-dependent PL spectra for $Cs_3Cu_2I_5$.
Fig. S10: *T*-dependent time-resolved PL (TRPL) decay lifetime.
Fig. S11: The electron spin resonance (ESR) measurement setup.
Fig. S12: ESR spectra of $Cs_3Cu_2I_5$ with 450 nm light irradiation.
Fig. S13: The X-ray photoelectron spectroscopy (XPS) measurement setup.
Fig. S14: XPS spectra of $Cs_3Cu_2I_5$ with/without 280 nm light irradiation.
Fig. S15: *T*-dependent magnetization in the excited and unexcited states of $Cs_3Cu_2I_5$.
Fig. S16: **B**-dependent energy splitting of $Cs_3Cu_2I_5$ at T = 30, 60K.
Fig. S17: Particle size distribution of $Cs_3Cu_2I_5$ grown at different temperatures.
Fig. S18: The PL spectra at T = 4.2 K of $Cs_3Cu_2I_5$ grown at different temperature.
Fig. S19: FWHM varies 40 K to 300 K for $Cs_3Cu_2I_5$ grown at different temperature.
Fig. S20: The energy splitting of $Cs_3Cu_2I_5$ thin films grown at 323 K and 373 K.
Fig. S21: Comparison of $Cs_3Cu_2I_5$ thin films samples grown at different temperatures.
Fig. S22: Electric and optical properties of the $Cs_3Cu_2I_5$ LED device.
Fig. S23: PL intensity and P_{PL} of $Cs_3Cu_2I_5$ films from −42 T to 42 T at different *T*.
Fig. S24: *T*-dependent *P* for both PL and EL at **B** = 15 T.
Fig. S25: *I*-*V* curves of the $Cs_3Cu_2I_5$ LED devices modulated by **B**.
Fig. S26: XRD pattern of $CsCu_2I_3$.
Fig. S27: SEM image of $CsCu_2I_3$.
Fig. S28: Elemental mapping and EDS spectrum of $CsCu_2I_3$.
Fig. S29: *T*-dependent normalize PL spectra of the $CsCu_2I_3$ films.
Fig. S30: Polarized PL spectra of $CsCu_2I_3$ films under 80 K, 180 K, and 300 K.
Supplementary Note1: Spin Hamiltonian analysis

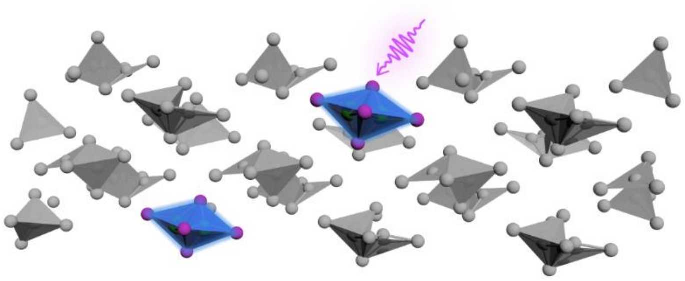

Figure S1. Schematic diagram of the J-T distorts occurs in $Cs_3Cu_2I_5$, where some collinear CuI_3 and CuI_4 units transform into two coplanar CuI_4 units upon UV excitation. The gray polyhedra denote unexcited $[Cu_2I_5]^{3-}$ clusters, while the blue polyhedra indicate the excited $[Cu_2I_5]^{2-}$ clusters, analogous to those observed in DMS materials.

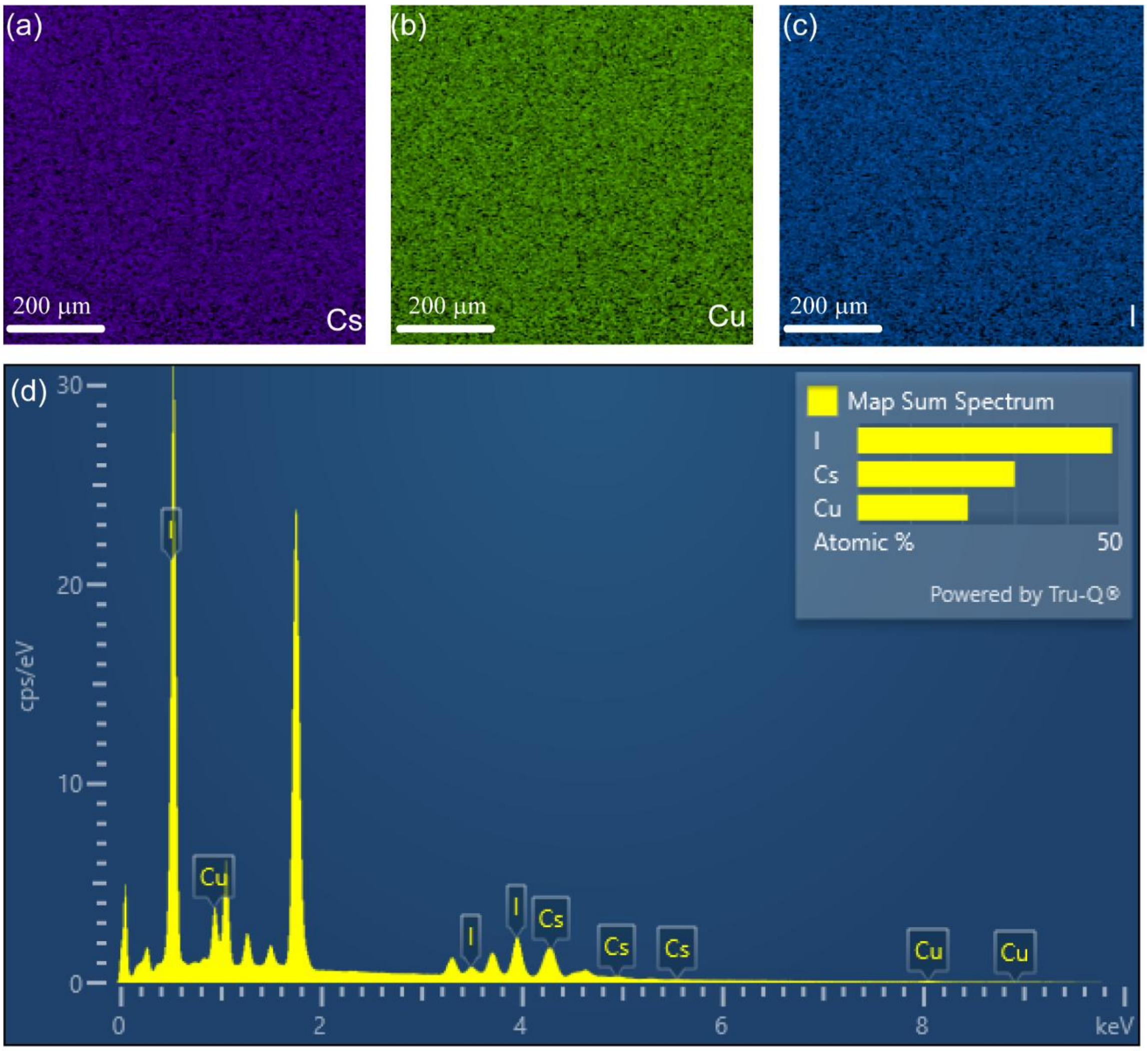

(e)

Map Sum Spectrum			
Element	Cs	Cu	I
Atomic (%)	30.17	21.13	48.70

Figure S2. (a-c) Elemental mapping and (d) energy dispersive X-ray spectroscopy spectrum (EDS) of the $Cs_3Cu_2I_5$ thin film, (e) atomic concentration of each element. The EDS data confirmed a uniform distribution of Cs, Cu, and I elements with an atomic ratio of 3.02:2.11:4.87, very close to the stoichiometric ratio of 3:2:5.

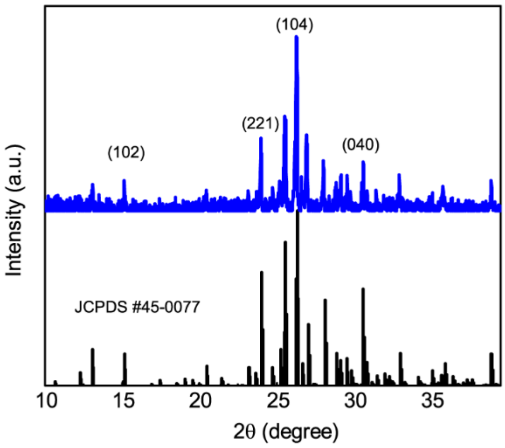

Figure S3. X-ray diffraction (XRD) patterns of the $Cs_3Cu_2I_5$ powder in comparison to the standard JCPDS#45-0077 with orthorhombic symmetry. The lattice constants, a = 10.04 Å, b = 11.67 Å, and c = 14.39 Å, are obtained from the XRD data.

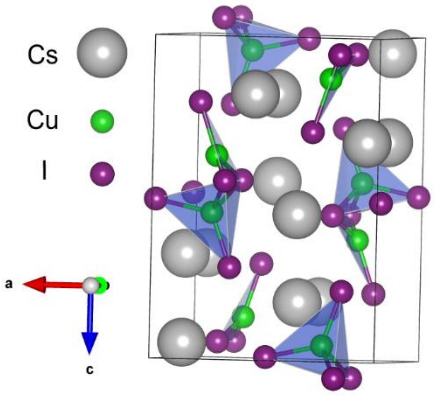

Figure S4. Atomic arrangements of $Cs_3Cu_2I_5$, where two edge-sharing Cu^+ structure components, $[CuI_4]^{3-}$ tetrahedral and $[CuI_3]^{2-}$ triangles, form the $[Cu_2I_5]^{3-}$ units represented by blue polyhedra. These $[Cu_2I_5]^{3-}$ units are isolated from each other and exhibit a 0D structural behavior.

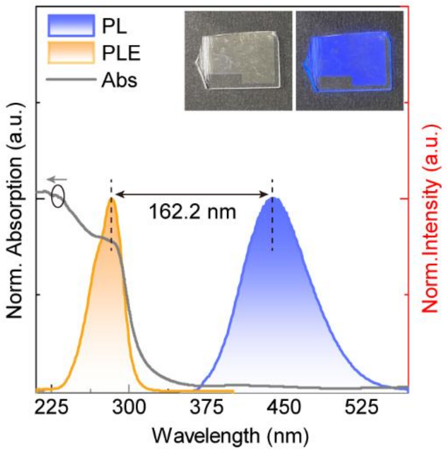

Figure S5. PL, PL excitation (PLE), and optical absorption spectra of the $Cs_3Cu_2I_5$ film, inset show the sample illuminated by white light and UV light, respectively. We have normalized the intensities of all three spectra to enable a direct comparison between the PL peak position and the absorption edge, revealing a Stokes shift of 162.2 nm, which is a characteristic feature of self-trapped exciton (STE) emission.

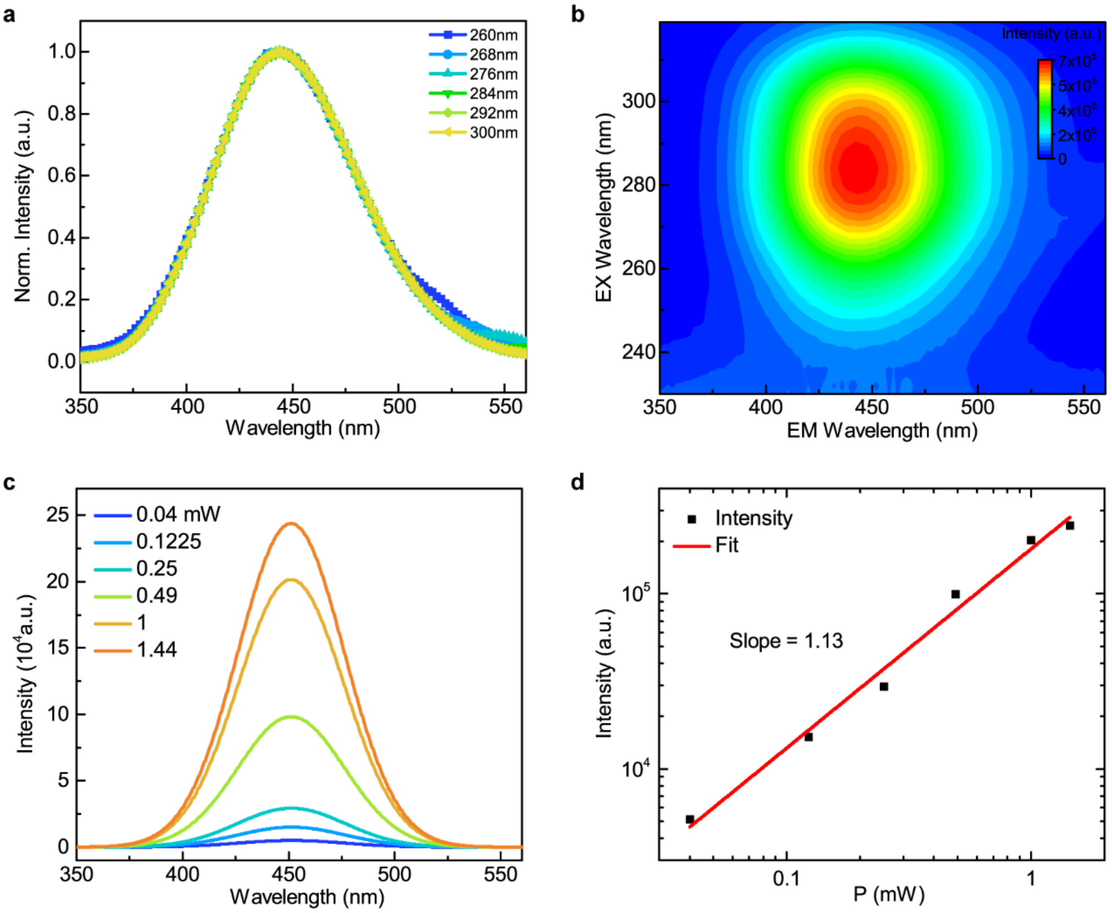

Figure S6. (a) Normalized excitation wavelength-dependent PL spectra of $Cs_3Cu_2I_5$ thin film. (b) Excitation-emission spectra with the intensity indicated by pseudo-color. (c) The excitation power-dependent PL spectra and (d) the corresponding PL intensity versus power, showing a slope close to 1, indicating excitonic emission.

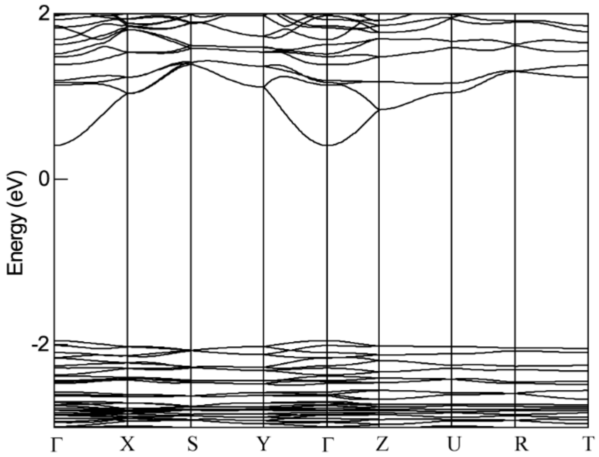

Figure S7. Band structure of $Cs_3Cu_2I_5$ from first-principles calculations. $Cs_3Cu_2I_5$ is a direct band gap semiconductor at the Γ point, and the flat valence band edge indicates the low dimensional nature of the material.

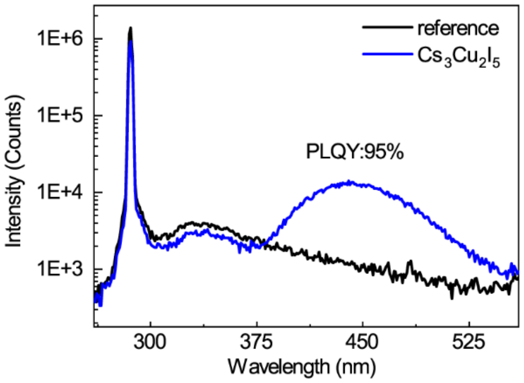

Figure S8. Photoluminescence quantum yield (PLQY) of $Cs_3Cu_2I_5$ under excitation at 285 nm.

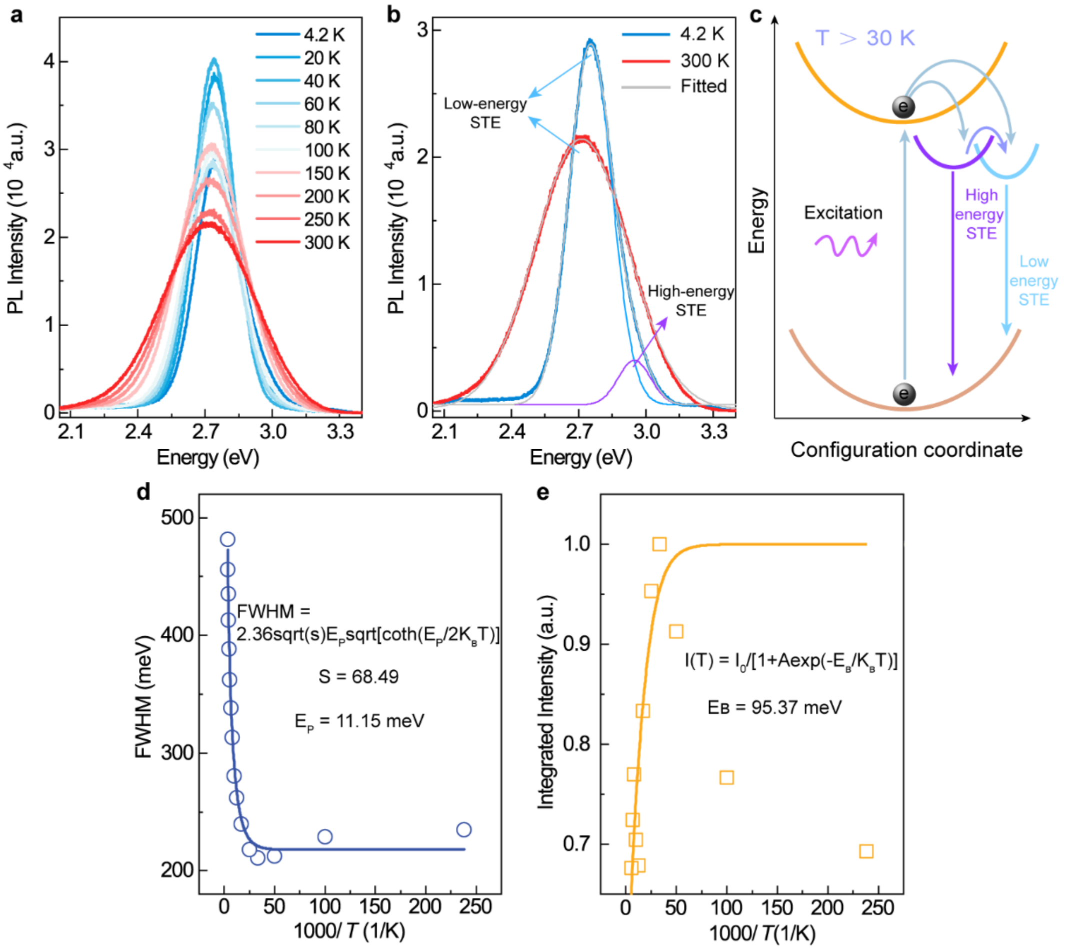

Figure S9. (a) Temperature-dependent PL spectra for $Cs_3Cu_2I_5$ thin film. (b) PL spectra of $Cs_3Cu_2I_5$ at 4.2 K and 300 K. The gray line represents the Gaussian fitting, while the cyan and purple solid lines correspond to the low-energy STE and high-energy STE emissions, respectively. (c) The configuration coordinate model of $Cs_3Cu_2I_5$ depicting the photophysical process above 30 K. (d) Temperature dependence of the full width at half-maximum (FWHM), The temperature dependence of the FWHM follows the relation[1]: $FWHM = 2.36\sqrt{S}E_p\sqrt{\coth\,[E_p/(2k_\mathrm{B}T)}$, where S is Huang-Rhys factors, E_p is the phonon energy, k_B is Boltzmann constant. By fitting the experimental data, the S is determined to be 68.49, and $E_p$is 11.51 meV. (e) PL intensity versus temperature. The temperature dependence of PL intensity can typically be described by the Arrhenius equation[2]: $I(T) = I_0/(1 + Ae^{-E_B/(k_\mathrm{B}T)})$, where I_0 represents the initial PL intensity (typically at 0 K), E_B is the activation energy of exciton thermal quenching. By fitting the experimental data, the exciton activation energy is determined to be 95.37 meV. Notably, at temperatures below 30 K, deviations from the fitting curve may arise from the emergence of high-energy STE emission. The suppressed phonon energy at low temperatures limits non-radiative recombination and relaxation pathways of these high-energy STEs, promoting radiative recombination and leading to detectable PL emission. This competing emission interacts with the primary low-energy STE, ultimately reducing its PL intensity.

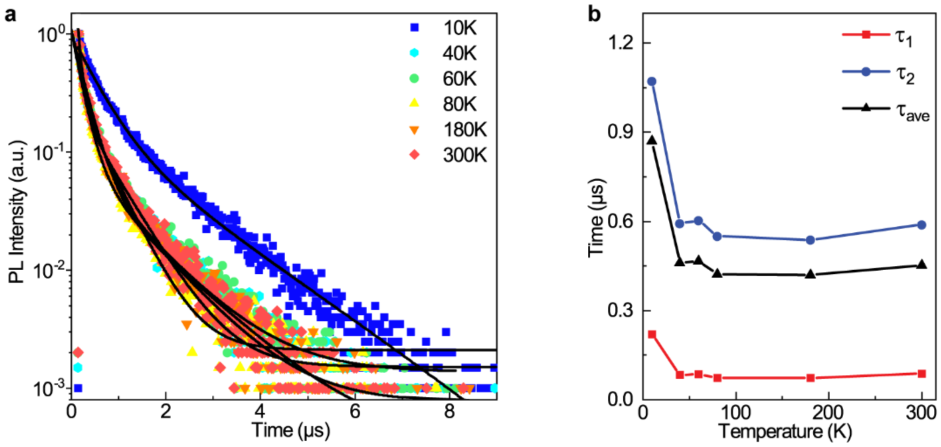

Temperature	A_1	τ_1 (μs)	A_2	τ_2 (μs)	τ_{ave} (μs)
10 K	0.596	0.218	0.404	1.063	0.596
40 K	0.709	0.071	0.291	0.547	0.432
60 K	0.669	0.069	0.331	0.527	0.431
80 K	0.716	0.062	0.284	0.498	0.394
180 K	0.724	0.070	0.276	0.537	0.418
300 K	0.704	0.077	0.296	0.562	0.443

Figure S10. (a) Time-resolved PL decay of the sample at different temperature, The black solid line represents the fitting curve (double exponential decay): $I = A_1\exp(-t/\tau_1) + A_2\exp(-t/\tau_2)$, where the τ_1 and τ_2 represent the fast and slow decay time, respectively, while A_1 and A_2 are the weighting factors of the corresponding decay components. (b) PL lifetime versus *T*. The average lifetime can be calculated using: $\tau_{ave} = (A_1\tau_1^2 + A_2\tau_2^2)/(A_1\tau_1 + A_2\tau_2)$. The table below summarizes the lifetime parameters obtained from biexponential decay fitting. Notably, a significant increase in lifetime is observed at 10 K, likely due to the suppression of non-radiative recombination as reduced phonon energy limits carrier relaxation pathways[3,4]. Additionally, this effect may be influenced by the emergence of high-energy STE emission at low temperatures.

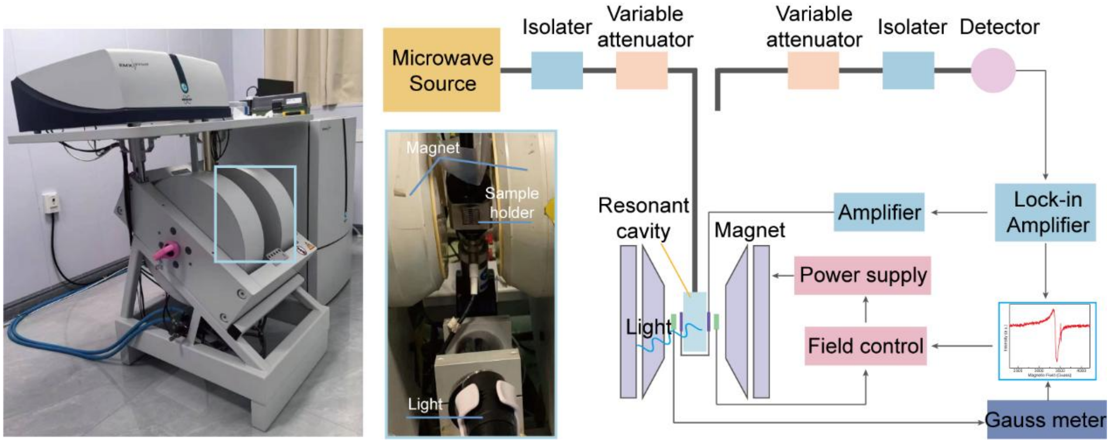

Figure S11 Schematic diagram of the electron spin resonance (ESR) measurement setup. The photon-excited ESR system integrates a conventional ESR setup with a tunable light source to investigate spin dynamics under optical excitation. This system comprises a microwave source, resonant cavity, tunable magnet, light source (e.g., laser or LED), detector, and data acquisition unit. The experimental procedure follows these steps: (i) Dark-state measurement: ESR signals of the sample are recorded in the absence of illumination to establish the baseline. (ii) Optical excitation: The sample is illuminated with a specific wavelength light source (280 nm) to excite charge carriers. (iii) Illuminated ESR measurement: After 5 minutes of continuous illumination, ESR spectra are recorded in real time as a function of the magnetic field. (iv) Data analysis: The variation in the g-factor and the light-induced spin-state modulation are analyzed to elucidate the underlying light-spin interactions. The measurement was conducted using a Bruker, A300-10/12 spectrometer.

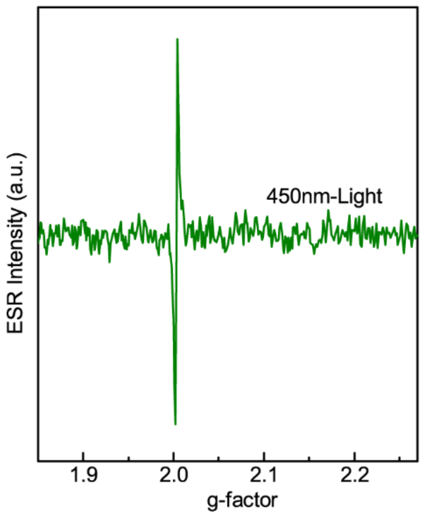

Figure S12. The electron spin resonance spectra under irradiation at the wavelength of 450 nm.

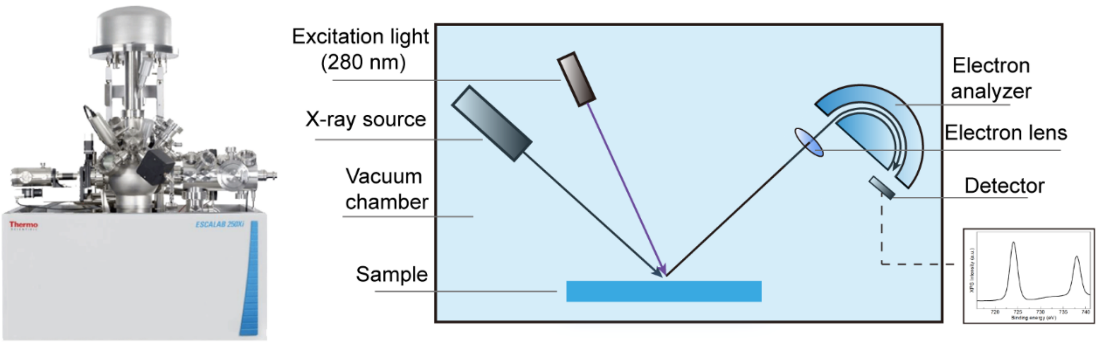

Figure S13 Schematic diagram of the X-ray photoelectron spectroscopy (XPS) measurement setup. The photon-excited XPS system incorporates a controlled light source into a conventional XPS setup, enabling direct investigation of surface electronic structures and chemical state evolution under illumination. The system comprises an X-ray source, an electron energy analyzer, an ultrahigh vacuum chamber, and a tunable light source—such as a monochromatic laser or LED—integrated via an optical delivery system. The experimental protocol involves three key steps: (i) dark-state measurements, where the $Cs_3Cu_2I_5$ sample is characterized in the absence of illumination; (ii) photoexcitation, in which the sample is irradiated with a 280 nm UV source for 30 min, followed by continuous exposure during XPS measurements; and (iii) data acquisition and analysis to resolve binding energy shifts, valence state transitions, and surface charge accumulation effects. The instrument model is Thermo Escalab 250Xi.

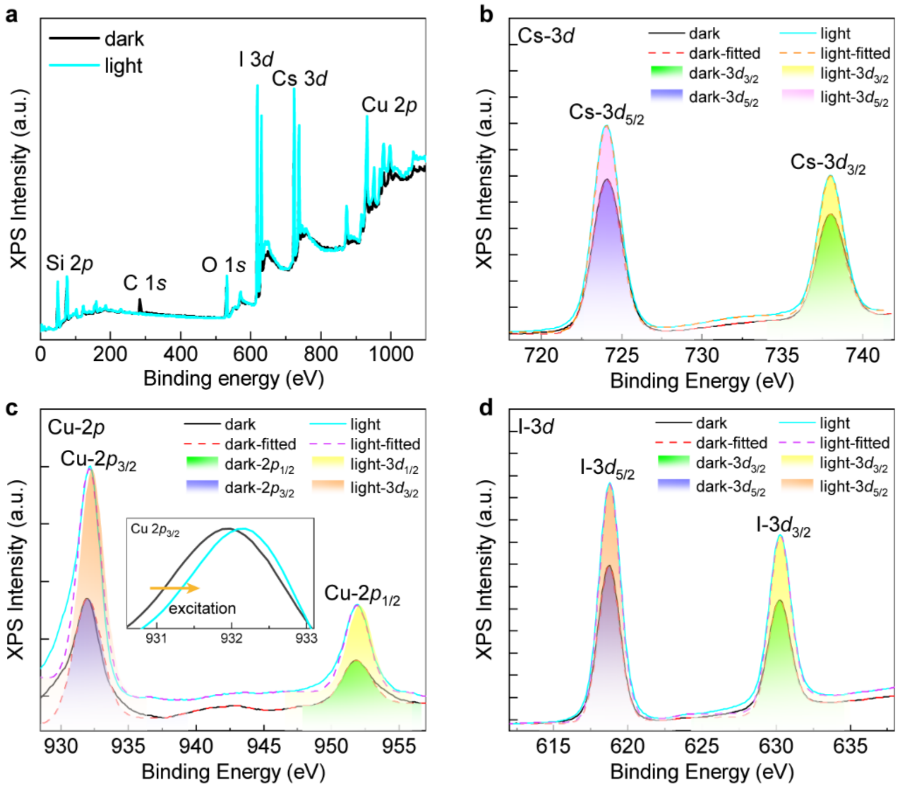

Figure S14. (a) X-ray photoelectronic spectroscope (XPS) pattern of the $Cs_3Cu_2I_5$ thin film with and without the illumination of 280 nm. High-resolution XPS element analysis shows (b) Cs-3*d*, (c) Cu-2*p* and (d) I-3*d*. Notably, the light generates heat on the sample, producing an etching-like effect that reduces the C signal after illumination. The Cs-3*d* spectrum exhibits two distinct peaks at 724 eV and 738 eV, providing conclusive evidence for the existence of Cs^+ in the sample. Likewise, the I-3*d* spectrum shows two distinct peaks at 618.8 eV and 630.3 eV, which confirms the I^- state. The inset of (c) shows the shift of the Cu-2$p_{3/2}$ characteristic peaks with UV-excitation, which signifies electron loss of outer electrons and provides support for the conversion in electron configuration from Cu^+ to Cu^{2+}.

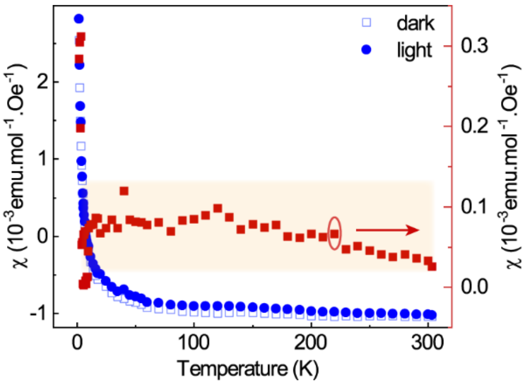

Figure S15. Temperature-dependent magnetic susceptibility in the excited and unexcited states of $Cs_3Cu_2I_5$. Blue hollow squares represent the dark state, blue solid circles represent the illuminated condition, and red solid squares indicate the difference in magnetic susceptibility with and without light. A significant enhancement in magnetic susceptibility under illumination is observed, with only a few hops occurring in the range of 4 K to 9 K.

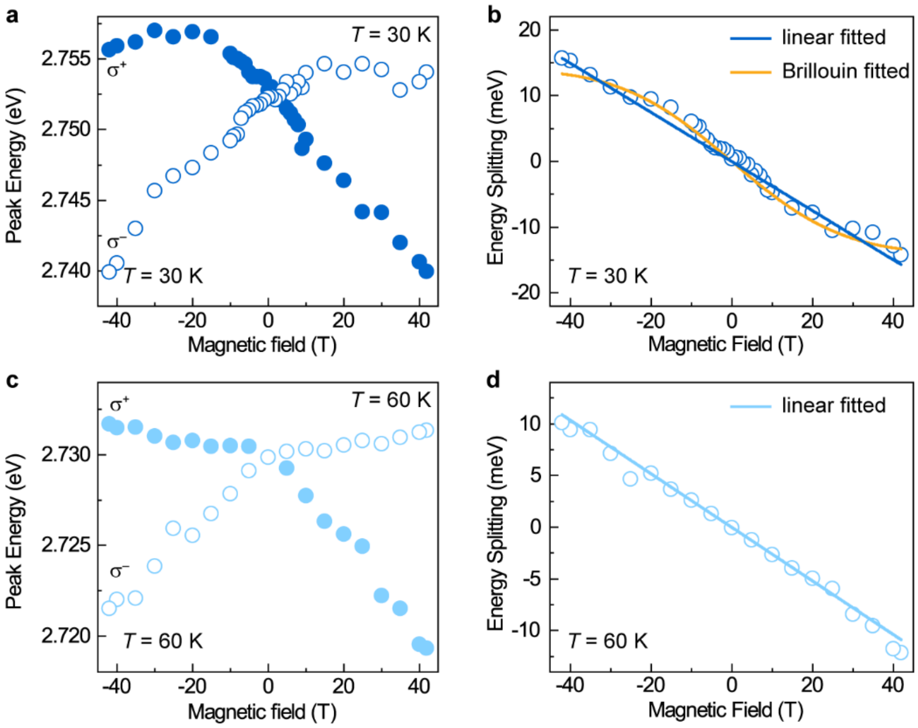

Figure S16. (a) At $T = 30$ K, the **B**-dependent peak energy of σ^+ and σ^-, and (b) the corresponding energy splitting, ΔE, which could not to be fitted with a linear fit but was well-fitted by the Brillouin function, indictating significant *sp-d* exchange interaction with a fitted exchange interaction energy of 16 meV. In addition, we obtained a geff of −8.27 in the low magnetic field region. (c) $T = 60$ K, the **B**-dependent peak energy of σ^+ and σ^-, and (d) the corresponding ΔE, which could be fitted by $\Delta E = g_{\mathrm{eff}}\mu_{\mathrm{B}}B$. The fitted g_{eff} value of −4.4 is in good agreement with the g_{ex} obtained from the fitting of P versus **B**. Although the effect of *sp-d* exchange interaction on ΔE diminishes as the T rises to 60 K, spin-polarized emission persists up to room temperature. The relatively large g_{ex} also suggests that *sp-d* exchange interaction may still influence the circularly polarized PL emission.

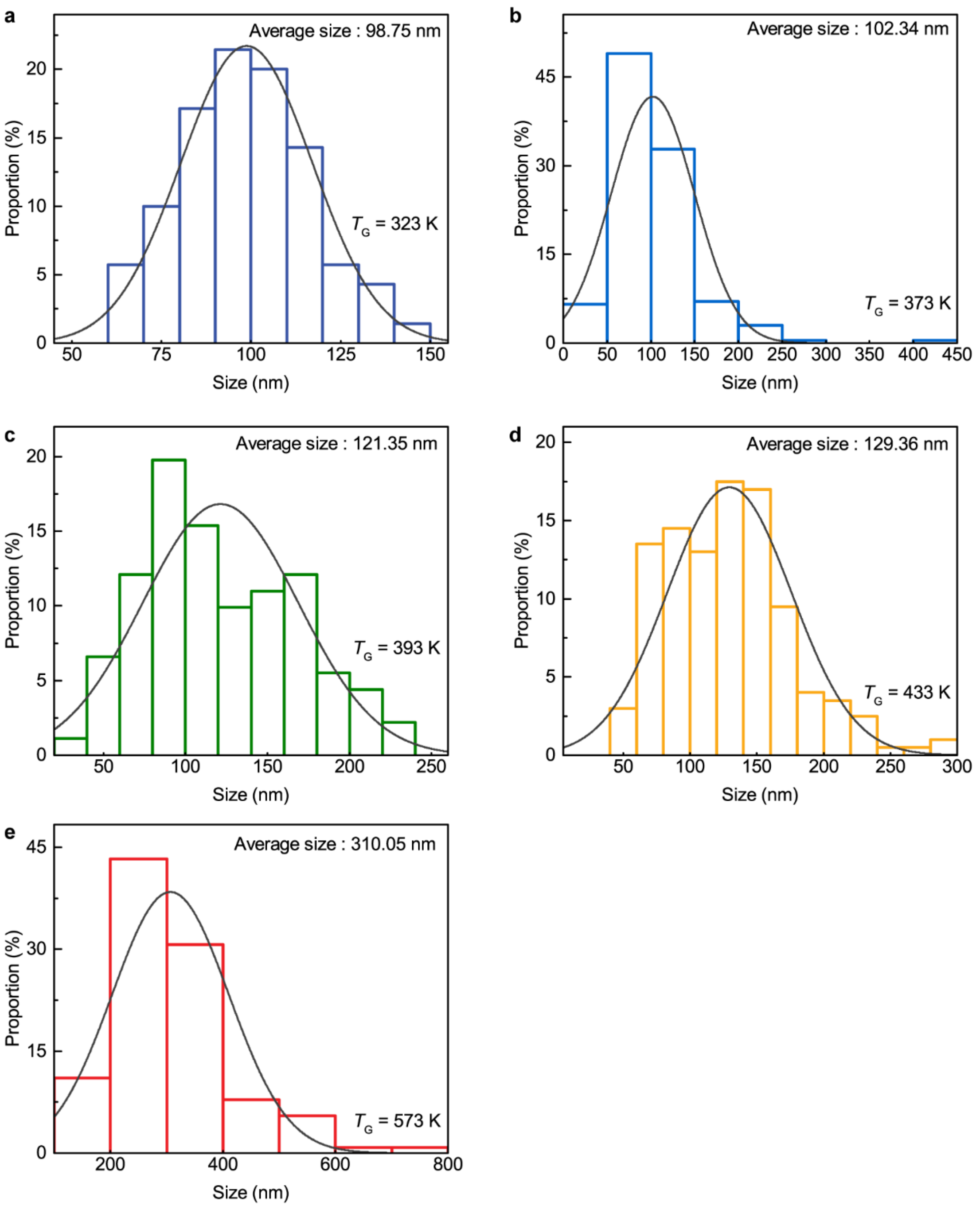

Figure S17. The distribution of particle size in the $Cs_3Cu_2I_5$ thin films with different growth temperatures (T_G) ranges from 323 K to 573 K.

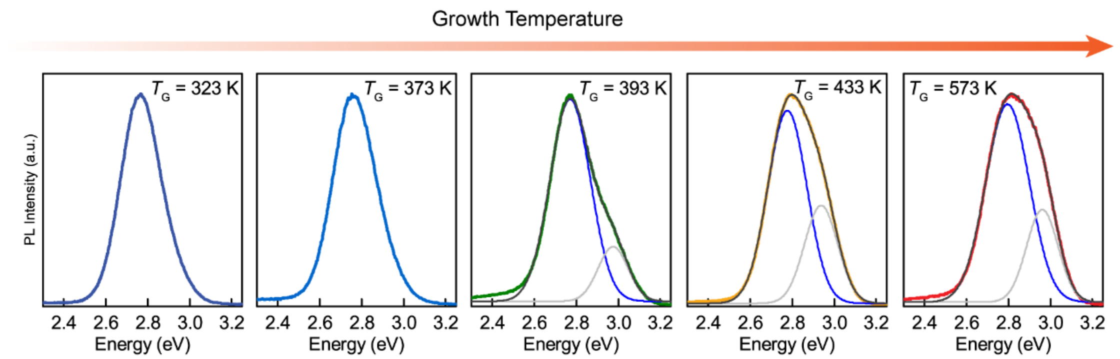

Figure S18. PL spectra measured at T = 4.2 K for the $Cs_3Cu_2I_5$ thin films with different T_G ranges from 323 K to 573 K.

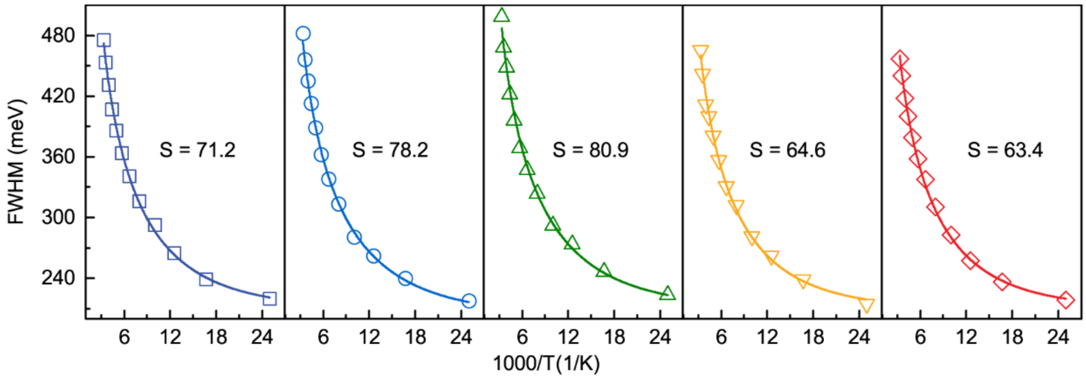

Figure S19. T_G-dependent FWHM measured at the temperatures of 40–300 K, solid lines are fit curves using $FWHM = 2.36\sqrt{S}E_p\sqrt{\coth[E_p/(2k_BT)]}$.

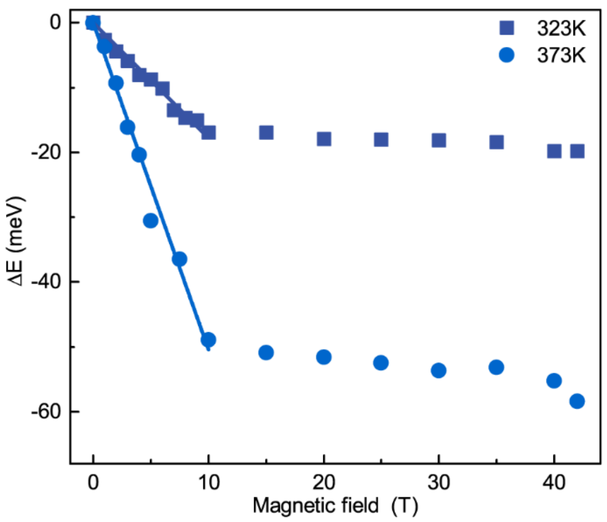

Figure S20. Magnetic field-dependent energy splitting (ΔE) for the $Cs_3Cu_2I_5$ thin films grown at T_G = 323 K and 373 K, respectively. Solid lines are fit curves using $\Delta E = g_{eff}\mu_B B$, through these fits, g_{eff} values are −30.7 and −93.5, for T_G = 323 K and 373 K, respectively.

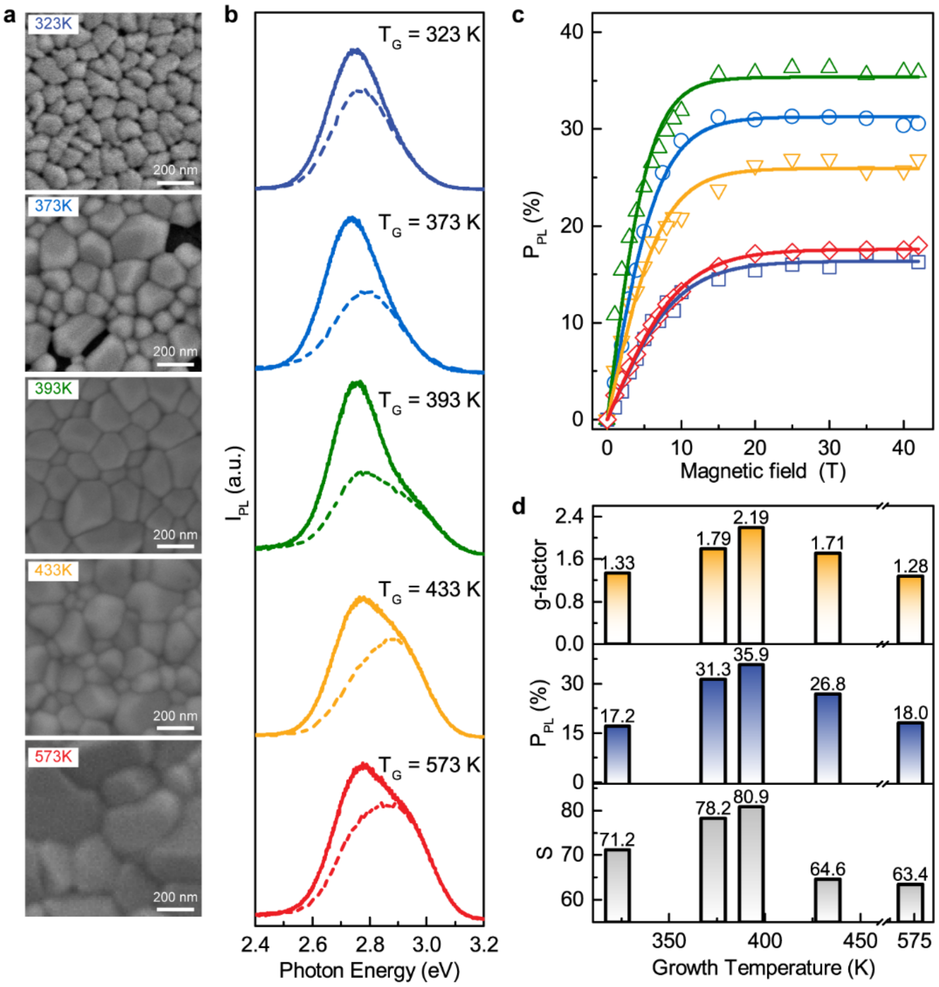

Figure S21. (a) Scanning electron microscope (SEM) images of $Cs_3Cu_2I_5$ films for different T_G. (b) σ^+-(solid line) and σ^--(dash line) polarized PL spectra for samples grown at T_G ranging from 323 K to 573 K, measured at T = 4.2 K and **B** = 42 T. (c) P versus **B** for different T_G. (d) T_G-dependent Huang-Rays factor (S, gray columns), maximal P (blue columns), and g_{ex}-factor (orange columns) from the fitting of the data in (c) using the modified-Brillouin function: $P = P_0 tanh\left[g_{eff}\mu_B B/\left(2k_B T\right)\right]$ in the main text.

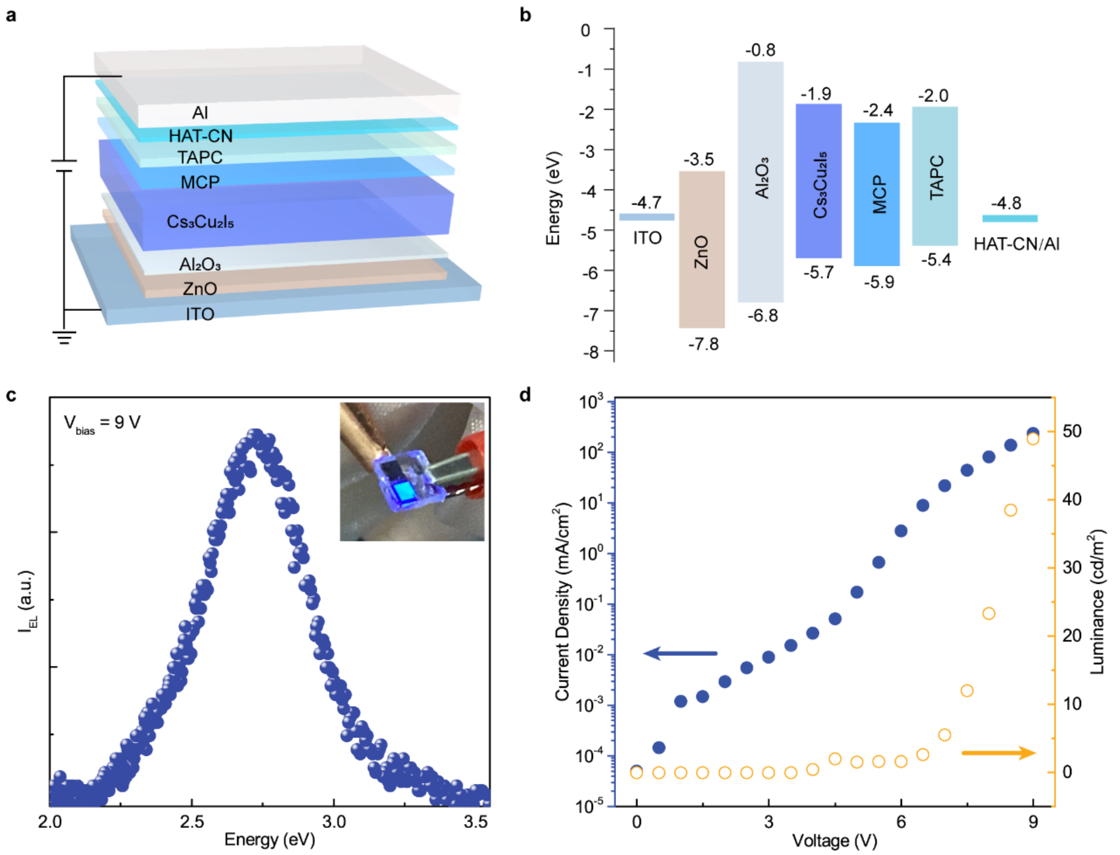

Figure S22. (a) Schematic illustration of the blue LED with a heterostructure of ITO/ZnO/Al_2O_3/$Cs_3Cu_2I_5$/MCP/TAPC/HAT-CN/Al. (b) Energy level diagram of the device with different layers. (c) EL spectrum of the device, inset shows the blue EL emission. (d) Current density and luminance versus voltage for the blue LED using $Cs_3Cu_2I_5$ thin film as the active region.

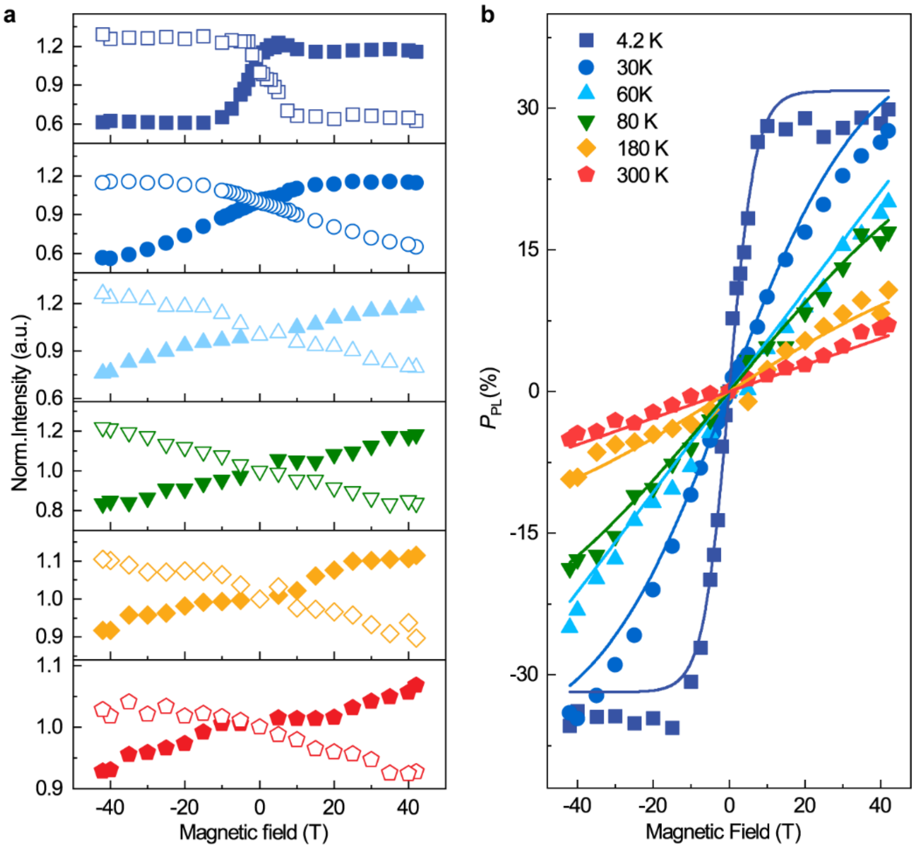

Figure S23. (a) **B** = 42 T, circular polarized PL intensity at T of 4.2 K,30 K,60 K,80 K,180 K, and 300 K. (b) The corresponding P_{PL} of the $Cs_3Cu_2I_5$ thin films. The excitonic g values were estimated using Brillouin function, the values are 2.47, 3.64, 4.01, and 3.97 at T = 30K, 80 K, 180 K, and 300 K, respectively.

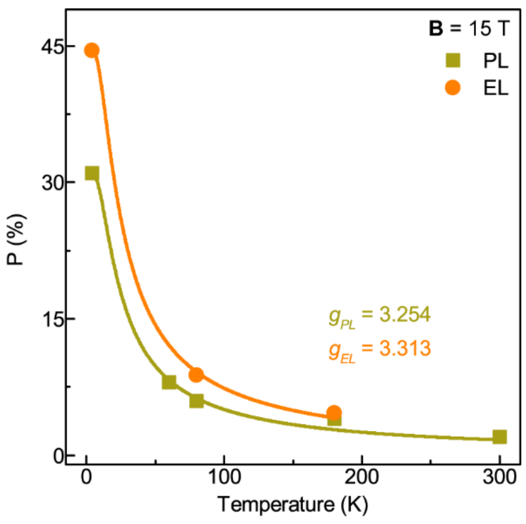

Figure S24. Temperature-dependent degree of circular polarization of both PL and EL at **B** = 15 T.

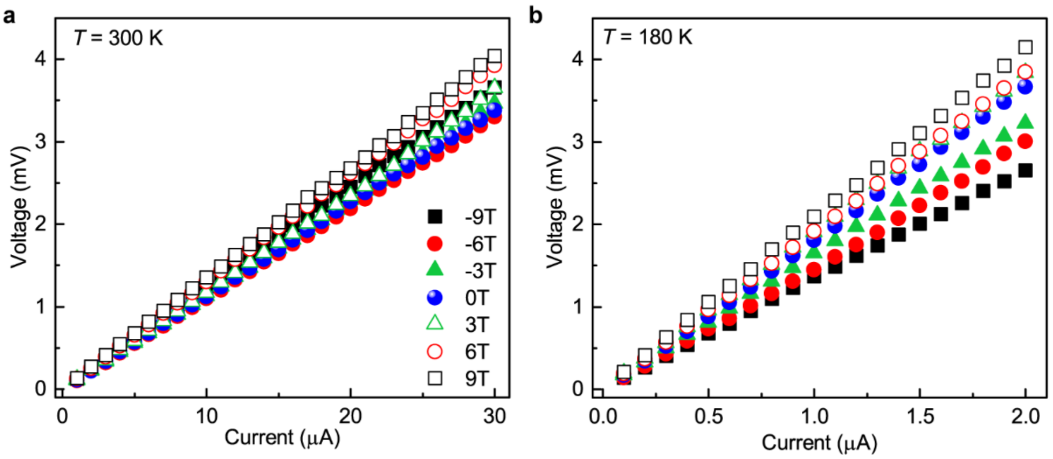

Figure S25. Current-voltage (*I*-*V*) curves of the device under different external magnetic fields measured at (a) *T* = 300 K and (b) *T* = 180 K. The *I*-*V* curves demonstrate a conspicuous magnetic field dependence, and the magnetic field effect on the electrical signals becomes stronger when *T* decreases from 300 K to 180 K, which is similar with P_{EL}. This spin-polarization control manifests as a linear shift and directional dependence in the *I*-*V* characteristics: under a positive magnetic field, parallel spin alignment enhances scattering, resulting in a high-resistance state and reduced current, whereas under a negative field, antiparallel spin alignment suppresses scattering, yielding a low-resistance state and increased current. Such magnetically induced resistance asymmetry reveals a strong coupling between spin degrees of freedom and charge transport in $Cs_3Cu_2I_5$, providing the fundamental physical basis for spintronic devices such as spin valves and memory units.

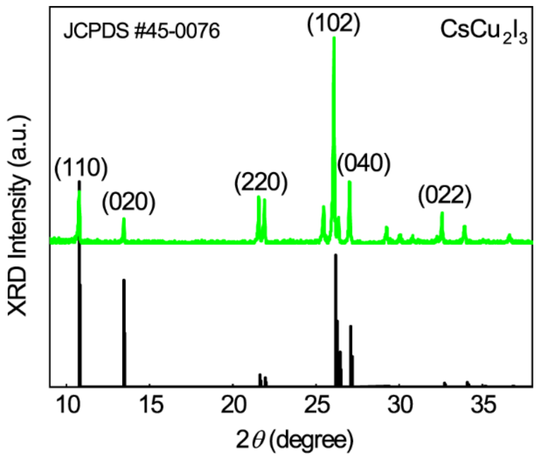

Figure S26. XRD pattern of $CsCu_2I_3$. The diffraction peaks at 10.77°, 13.46°, 21.64°, 26.19°, 27.11°, and 32.54° correspond to the (110), (020), (220), (102), (040), and (022) planes of the orthorhombic $CsCu_2I_3$ phase (JCPDS #45-0076), confirming the successful synthesis of orthorhombic $CsCu_2I_3$ with the Cmcm space group.

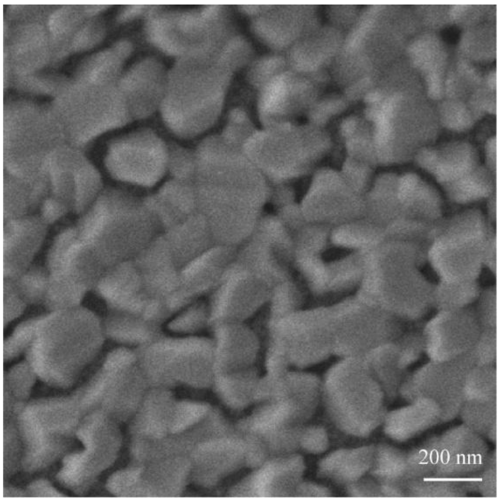

Figure S27. SEM image of $CsCu_2I_3$. The film exhibits a uniform grain size distribution with few voids, indicating the synthesis of a high-quality $CsCu_2I_3$ thin film.

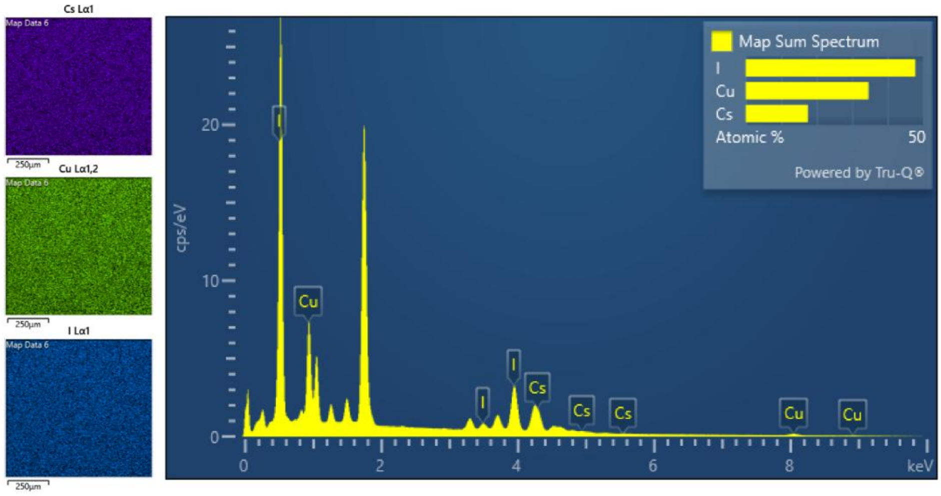

Figure S28. Elemental mapping and EDS spectrum of $CsCu_2I_3$. The measured elemental composition of Cs, Cu, and I is 17.55%, 34.59%, and 47.86%, respectively, aligning well with the expected stoichiometry of $CsCu_2I_3$. The elemental distribution maps further reveal a uniform spatial distribution of Cs, Cu, and I throughout the sample, confirming the successful synthesis of high-quality $CsCu_2I_3$ thin films.

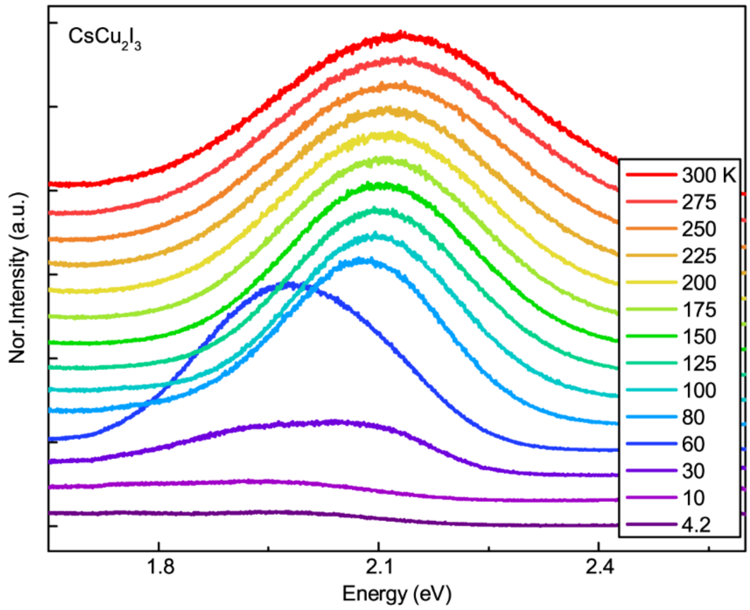

Figure S29. *T*-dependent PL spectra of $CsCu_2I_3$ films from 4.2 K to 300 K. The investigation revealed luminescence quenching in $CsCu_2I_3$ at T < 60 K, which can be attributed to a structural phase transition. In order to augment comparability for $CsCu_2I_3$ and $Cs_3Cu_2I_5$, the PL spectra within the temperature range of 80 K to 300 K are chosen for the FWHM analysis.

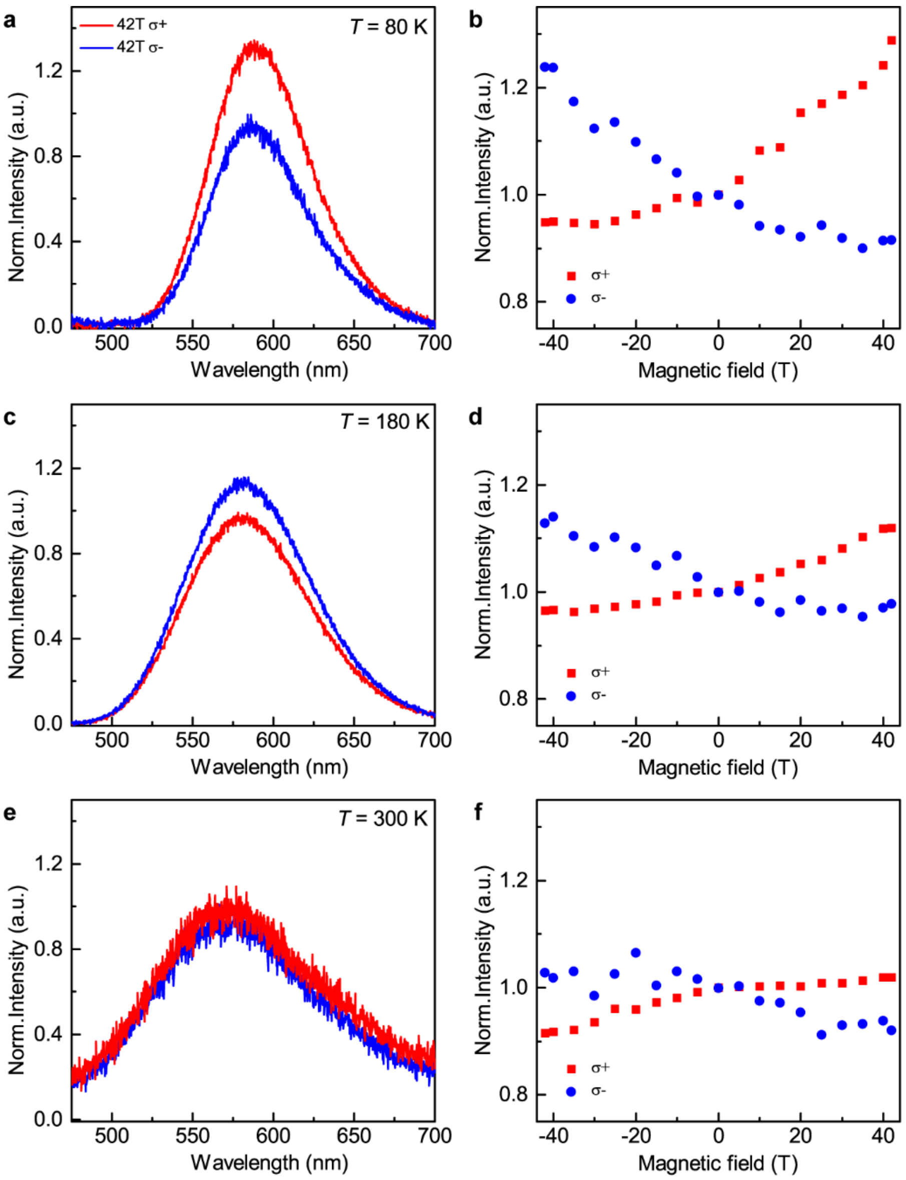

Figure S30. At **B** = 42 T, the σ^+, σ^- PL spectra of $CsCu_2I_3$ films at the temperatures of (a) 80 K, (c) 180 K, and (e) 300 K, and the corresponding σ^+ and σ^- PL peak intensities of $CsCu_2I_3$ films under magnetic fields range from −42 T to 42 T of (b) 80 K, (e) 180 K, and (f) 300 K, respectively.

Supplementary Note1:

The spin Hamiltonian analysis is performed to determine possible microscopic structure and chemical origin of the defect responsible for the photo-active ESR signal. The effective spin Hamiltonian takes the form of $\mathrm{H} = \mu_{\mathrm{B}}\boldsymbol{B}\cdot\mathbf{g}\cdot\boldsymbol{S} + \boldsymbol{S}\cdot\boldsymbol{A}\cdot\boldsymbol{I}$, where μ_{B} is the Bohr magneton, $\mathbf{B}$ is an external magnetic field, $\mathbf{A}$ is hyperfine (hf) tensor. We note that though a ligand field and the spin-orbit interaction are not explicitly included in the spin Hamiltonian, they are reflected by the $\mathbf{g}$ and $\mathbf{A}$ tensor anisotropy, that depend on orientation of ligand field and orbital character of the *d*-electron electronic states. This anisotropy is used to determine the local symmetry of the defect being probed by the ESR.

References

1. Hui, Y., Chen, S., Lin, R., Zheng, W. & Huang, F. Photophysics in $Cs_3Cu_2I_5$ and $CsCu_2I_3$. *Mater. Chem. Front.* **5**, 7088-7107 (2021).
2. Jun, T. *et al.* Lead-Free Highly Efficient Blue-Emitting $Cs_3Cu_2I_5$ with 0D Electronic Structure. *Adv. Mater.* **30**, e1804547 (2018).
3. Kirchartz, T., Markvart, T., Rau, U. & Egger, D. A. Impact of Small Phonon Energies on the Charge-Carrier Lifetimes in Metal-Halide Perovskites. *J. Phys. Chem. Lett.* **9**, 939-946 (2018).
4. Lian, L. *et al.* Photophysics in $Cs_3Cu_2X_5$ (X = Cl, Br, or I): Highly Luminescent Self-Trapped Excitons from Local Structure Symmetrization. *Chem. Mater.* **32**, 3462-3468 (2020).
